# Nanostructured Coatings: Review on Processing Techniques, Corrosion Behaviour and Tribological Performance

**DOI:** 10.3390/nano12081323

**Published:** 2022-04-12

**Authors:** Sheikh Aamir Farooq, Ankush Raina, Sanjay Mohan, Ramachandra Arvind Singh, Subramanian Jayalakshmi, Mir Irfan Ul Haq

**Affiliations:** 1 School of Mechanical Engineering, Shri Mata Vaishno Devi University, Katra 182320, Jammu and Kashmir, India; sheikhaamirfarooq@gmail.com (S.A.F.); ankush.smvd@gmail.com (A.R.); sanjaysmvdu@gmail.com (S.M.); 2 School of Mechanical and Electrical Engineering, Wenzhou University, Wenzhou 325035, China

**Keywords:** nanostructured coatings, nanomaterials, ceramics, metallic, nanocomposites, synthesis, corrosion, tribology

## Abstract

Corrosion and tribology are surface phenomena. Modifying surfaces of materials without resorting to altering their bulk properties is an effective route to alleviate corrosion, friction and wear, encountered in engineering applications. With the advancements in the field of nanotechnology, surface protective coatings with nanomaterials can be readily developed to explore their functionality in mitigating chemical/physical damage of surfaces. Surface protection enhances performance and operating lifetimes of industrial machinery components. This review presents insights on various types of recently developed nanostructured coatings, their synthesis routes, corrosion behaviour and tribological performance. It provides the state-of-the-art information on the development of nanostructured coatings, namely, ceramic coatings, metallic coatings and nanocomposite coatings with metal and polymer matrices. Biomimetic approaches in making nanostructured coatings and challenges encountered in the development of nanostructured coatings are highlighted.

## 1. Introduction

Nanotechnology deals with the development and application of materials having size < 100 nm [1,2]. Since the early inception of nanotechnology in the 1980s [3], research, development and application of nanomaterials have exponentially progressed. By definition, a nanomaterial is a material having at least one of its dimensions in nanometre scale in three-dimensional space [4]. Due to their diminutive size, nanomaterials have high surface area to volume ratio, and so can provide chemical/physical properties not offered by micro/macro-sized materials. Owing to this fact, nanomaterials have found their niche in several applications, such as optical [5], thermal [6], chemical [7], mechanical [8] magnetic [9] and energy conversion/storage applications [10].

In industrial systems, corrosion and tribological issues of friction and wear adversely influence the performance and operating lifetime of components. For these reasons, huge energy and material losses occur in engineering equipment, which leads to enormous economic loss. This has propelled the design of various strategies towards the development of anticorrosion and antifriction/wear surface protection [11,12,13]. One such efficient and proven strategy is the application of coatings [13,14]. The design and development of coatings for a particular substrate, for a particular application and for a particular operating environment is a challenge, mainly due to numerous influencing parameters involved such as thickness, grain size, adhesion of coating with substrate, hardness, etc. [15,16]. Therefore, understanding the physical and chemical phenomena for a coating–substrate system is complex and interdisciplinary as it involves materials science, solid mechanics and electrochemistry [17]. In service, coatings experience mechanical loads and thermal stresses, which cause the formation of micro-cracks and consequently lead to their fracture/peeling off from their substrates. Apart from varying mechanical loads, coatings in real-life applications are exposed to a wide range of temperatures, such as up to 2700 °C for IC engines [18], 80–200 °C for solar applications [19], 2100 °C for gas turbines [20] and 100–300 °C for aerospace components [21]. This affects the service life of components, as the underlying substrate materials are directly exposed to their surrounding corrosive environment and are degraded [22], or, in the case of tribological conditions, substrate materials come in direct contact with their counter face, causing increased friction and wear.

The unique properties offered by nanomaterials in comparison to micro-sized materials have led to the successful development of nanostructured coatings, which have at least one of their constituents in nanometre scale. Nanostructured coatings provide enhanced surface protection, and hence are being utilised in the fields of electronics, medicine, food packaging, shipping, etc. [23,24].

This paper presents a review on the various aspects of recently developed nanostructured coatings that provide enhanced protection against corrosion and tribological issues. The review includes: (i) a brief description of various synthesising methods of nanostructured coatings; (ii) corrosion and tribological behaviour of nanocoatings, particularly metallic, ceramic nanostructured coatings and nanocomposite coatings with metal and polymer matrices; and the effect of parameters, such as grain size, composition, synthesis method, environment, additives, etc., on their corrosion and tribological behaviour; (iii) biomimetic approaches in developing advanced nanostructured coatings; (iv) challenges associated in the development of nanostructured coatings; and (v) summary and recommendations. Nanostructured coatings are referred to as “nanocoatings” in the ensuing sections.

## 2. Synthesising Methods

**Figure 1:**
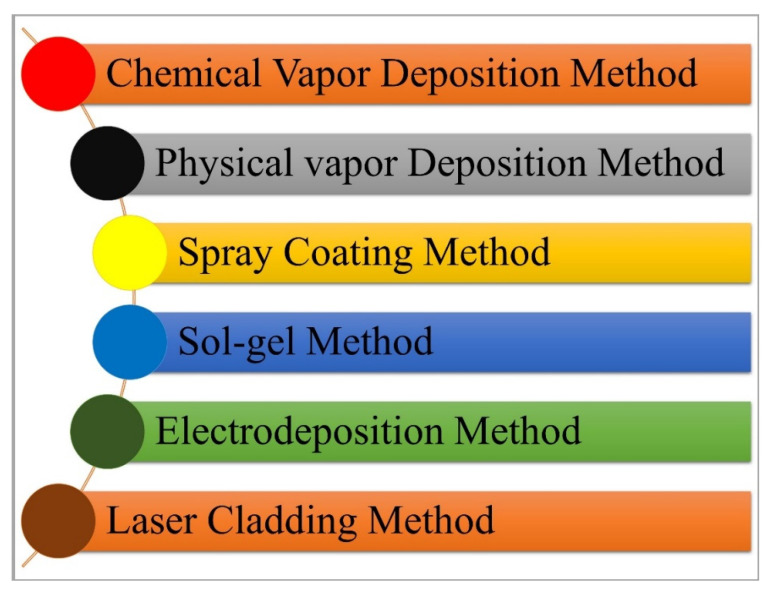
Different methods to produce nanostructured coatings.

Depending on the target application, different synthesis methods are used to produce nanocoatings. Besides traditional methods to produce nanostructured coatings, such as physical vapor deposition and chemical vapor deposition, novel approaches are also used to produce nanostructured coatings, such as laser cladding, the sol-gel method, etc. [23]. Different methods to produce nanostructured coatings are listed in Figure 1. During the application of coatings, it is also required to prepare substrates, which involves steps such as cleaning and chemical modification, as the coating quality on a substrate depends on the condition of the substrate surface such as cleanliness (absence of contaminants, dirt, oxides/scales), surface defects (pores, scratches), roughness, etc. [25]. Brief descriptions of the coating methods listed in Figure 1 are given below.

### 2.1. Chemical Vapor Deposition

The chemical vapor deposition (CVD) method involves dissociating gaseous or vaporous material by chemical reactions near a heated substrate surface, followed by the deposition of the coating on the heated substrate surface. This method is commonly used to coat metallic or ceramic compositions. Substrates are usually metallic or ceramic materials. The temperature of the substrate is crucial, as it can influence the adhesion of the coating to the substrate [26]. In the CVD method, multidirectional deposition of coating material on a substrate is possible, due to the inherent nature of the method [27]. This means that substrates having complex geometries and varying sizes can be readily coated using the method. CVD is used in a variety of industrial applications, including in the deposition of refractory materials on turbine blades. The CVD approach necessitates higher substrate temperatures, and a slow rate of precipitation, making it expensive for coating large surfaces. CVD methods are sub-classified based on how the chemical reaction is initiated. These include techniques such as atmospheric pressure CVD, low-pressure CVD, laser CVD, plasma-assisted CVD, plasma-enhanced CVD, atomic layer deposition, etc. [28].

### 2.2. Physical Vapor Deposition

The physical vapor deposition (PVD) method involves converting the target material to a gaseous phase and its subsequent deposition on a substrate surface. The target material is converted into the gaseous phase by the use of thermal energy, laser energy, high energetic particles or ions, electron beam, resistive heating, etc. [29], unlike in CVD, where a chemical reaction occurs between the gaseous substance and the substrate. The steps involved in the PVD method are (i) target material evaporation, (ii) transportation of evaporated material to substrate surface and (iii) deposition of target material as a coating on substrate surface [30]. PVD methods are distinguished by their principle of deposition of the coating material [23]. Examples of deposition methods are thermal evaporation, cathodic arc deposition, magnetron sputtering and laser pulsed deposition [28,31]. Novel fullerene-like, carbon-based nanostructured coatings such as fullerene-like carbon nitride (FL-CN_x_) and fullerene-like carbon fluoride (FL-CF_x_) nanostructured coatings formed via magnetron sputtering exhibit low water absorption due to the lack of dangling bonds, compared to conventionally used amorphous carbon films. Hence, FL-CN_x_ coatings are suitable protective overcoats for magnetic storage devices such as computer disk drive systems [32,33,34].

Experimental factors, i.e., processing parameters, play a crucial role in determining the structure of nanostructured coatings, which in turn influences and alters mechanical, wear and corrosion properties. For example, the microstructure, mechanical stress, roughness, hardness and wear performance of titanium–aluminium–nitride (Ti-Al-N) films formed via reactive magnetron sputtering are greatly influenced by substrate bias. For Ti-Al-N films, an increase in substrate bias leads to an increase in hardness and residual stress, whereas surface roughness and wear rate decrease [35]. Similarly, the dynamic glancing angle in DC magnetron sputtering affects the deposition rate, hardness and wear performance in Cr-Al-N nanocoatings. Dynamic Glancing Angle Deposition (DGLAD) is a method in which substrates are oscillated in front of targets with constant changes in angle. For example, an oscillatory range of ±10◦ results in around three times more wear-resistant coatings as compared to conventionally deposited coatings [36]. Due to the physical vapor deposition principle in the PVD method, the coating of substrates is limited to line-of-site deposition. By the PVD method, all forms of inorganic and some forms of organic materials can be deposited on substrates. Compared to other types of deposition techniques such as electroplating, PVD is environmentally friendly. However, PVD requires a vacuum chamber, which limits the dimensions of surfaces to be coated. Unlike the CVD method, substrates having intricate/complex surface geometries cannot be uniformly coated by the PVD process.

### 2.3. Spray Coating

Spray coating is a technique in which a target material is passed via a nozzle and deposited on a substrate surface by impact to produce a coating [37]. The sprayed substance can be either in form of liquid, molten or softened particles [26]. Spray coating techniques are usually used in industry to coat irregularly shaped glass, metals, wood and textiles with organic lacquers and binders. Thermal spraying is one of the most commonly used spray coating method. In this process, the coating material is melted or heated and sprayed onto the substrate. The coating material is usually heated by electrical or chemical processes. Substrates with large surface areas can be coated at higher deposition rates with thermal spraying compared to other conventional methods such as CVD and PVD. Cold spraying is a variation of spray coating which aids in the application of coatings at temperatures lower than those set in thermal spray methods. However, high velocities are required in cold spraying, as the impingement velocity influences the bonding between coating material and substrates. Cold-sprayed coatings have improved strength, low porosity and better adhesion capability [31]. Other types of spray coating methods are plasma spraying, high-velocity oxy-fuel spraying (HVOF), high-velocity air fuel (HVAF), etc. In these processes, the final properties of the sprayed coating depend on parameters such as size distribution of coating powders, powder feed rate, distance between spray gun and substrates, spray velocity, etc.

### 2.4. Sol-Gel Process

The sol-gel method involves the use of a solvent containing a chemically active component as a precursor, followed by hydrolysis and polycondensation to generate a sol system. A sol is slowly polymerised to form a gel, which is then dried and heated to form a coating [23,38]. The sol-gel method produces coatings with uniform chemical and physical properties. The coating produced by the sol-gel method depends on factors such as catalyst nature, initial material to produce sol, temperature, thickness, pH, etc. These factors affect the speed of hydrolysis and coating density. The sol-gel process can be used to produce wear-resistant coatings [23,39,40]. The sol-gel method has also been used to develop nanocomposite coatings with micron thickness. Coatings by the sol-gel method have thickness constraints and are susceptible to cracking [31]. Gels have micropores, and so gases/organics escape during the drying process, causing shrinkage. There are some disadvantages of sol-gel processes, especially when extended for large-scale production (i.e., industrial production): (i) the sol-gel process is time-consuming and usually requires long time duration (days or even weeks) for preparation, (ii) the raw materials needed in the sol-gel process are expensive and (iii) some organics used in the sol-gel process are hazardous to human health and the environment [23].

### 2.5. Electrodeposition

Electrodeposition involves the deposition of a metal or alloy coating material using an electric current (electrolysis) over a conductive substrate surface that is immersed in an electrolyte containing a salt of the metal or alloy that is to be coated. The electrolyte that contains the salt of the metal to be deposited is known as the bath. The composition, morphology and texture of the film coating can be altered by adjusting experimental parameters such as applied potential, current density, deposition time and composition of plating solution [41,42]. Electrodeposition is one of the most widely used methods for the production of nanostructured metallic or alloy coatings. Based on the type of the current used, electrodeposition is sub-classified as direct current (DC) electrodeposition and pulse electrodeposition.

### 2.6. Laser Cladding

Laser cladding is a method of depositing nanostructured coatings by melting powdered or wire feedstock target material using a high-energy laser on the surface of the substrate [23,43]. Laser process parameters such as laser power, power feed and scan speed have a significant influence on coating quality [44,45,46]. Laser cladding offers advantages such as high cooling rates, low dilution rates and ease of automation compared to other methods. Varieties of powders can be used to deposit coatings by this method. However, poor metallurgical quality, non-uniform composition and cracks in coatings are some of the disadvantages of the laser cladding process [23]. 

**Figure 2:**
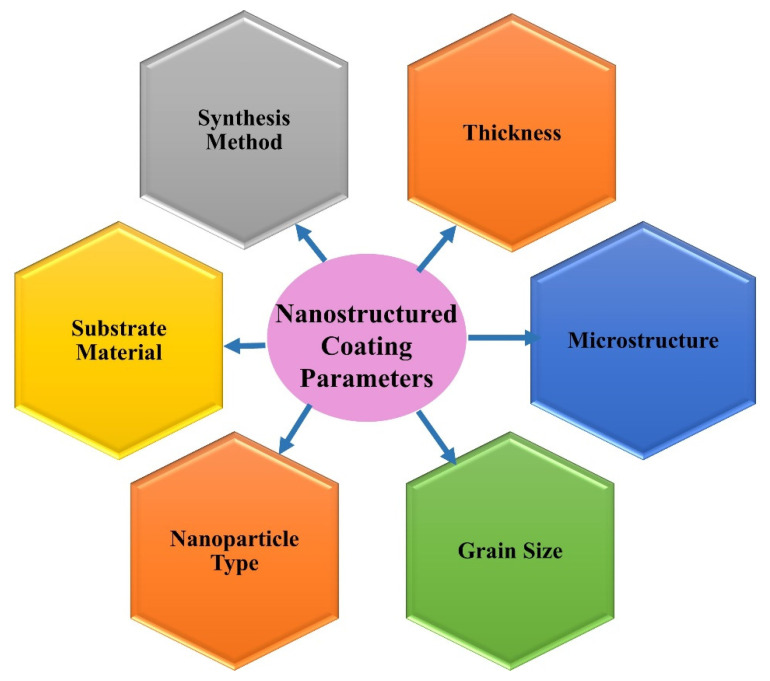
Various parameters influencing nanostructured coating formation.

Besides the above-mentioned methods to produce nanocoatings, other approaches to synthesise nanocoatings are layer-by-layer assembly, electroless deposition and self-assembly coatings [31,47,48,49]. Figure 2 shows the various parameters that are critical in the formation of nanocoatings. Table 1 summarises the coating preparation methods along with their process parameters.

## 3. Corrosion Behaviour

Corrosion causes the degradation of surfaces, which in turn causes component failures, eventually leading to the breakdown of machinery. At times, corrosion has led to catastrophic failures of industrial/domestic equipment, causing fatal accidents and environmental hazards [50,51]. Worldwide, economic losses due to corrosion are enormous due to material losses, equipment damage, repairs, maintenance costs and decreased operating efficiency of machinery [52]. The National Association of Corrosion Engineers (NACE) reports that global losses are estimated to be around USD 2.5 trillion, or ~3.4% of global gross domestic product (GDP) [53]. Although microcoatings provide protection against corrosion, imperfections in these coatings lower their effectiveness [54]. With the advent of nanomaterials and the development of nanocoatings, combating corrosion has become relatively more effective. Nanocoatings have multi-functionality. They can resist corrosion, temperature fluctuations, abrasion, adhesion, friction, fogging and can be biocompatible and anti-bacterial [27,55,56,57]. 

### 3.1. Ceramic Nanostructured Coatings

Ceramic nanocoatings are widely used in many applications such as engine valves, boiler parts, automotive body parts, orthopaedic implants, etc., due to their excellent resistance to corrosion, oxidation and wear, as compared to metals, especially in high-temperature applications. They also have excellent thermal and electrical insulation properties [58]. Ceramic materials and their application as nanocoatings are described in this section. 

#### 3.1.1. Alumina (Al_2_O_3_) Nanostructured Coatings

Alumina (Al_2_O_3_) is popular as ceramic coating material due to its excellent inherent resistance to corrosion and mechanical abrasion, and low electrical/thermal conductivity [59,60,61].

The synthesising route of Al_2_O_3_ nanocoatings has an influence on their anticorrosion performance. This is demonstrated in Ref. [62], where a comparison of the performance of nanocoatings produced by plasma-enhanced atomic layer deposition (ALD) with those produced via thermal-enhanced atomic layer deposition was made [62]. Al_2_O_3_ nanocoatings with thickness ranging from 10 to 50 nm were deposited on 100Cr6 steel and Al2024-T3 aluminium substrates. Nanocoatings produced by plasma-enhanced ALD were less porous due to their better film nucleation compared to thermal-enhanced ALD nanocoatings. It was observed that 10 nm thick nanocoatings produced by plasma-enhanced ALD remained intact over the substrates, whereas 10 nm thick nanocoatings produced by thermal-enhanced ALD showed poor adhesion and were detached from the substrates. Furthermore, it was identified that thickness of the nanocoatings affected their quality. Among the Al_2_O_3_ nanocoatings produced by both ALD techniques, the 50 nm thick nanocoatings were found to be the least porous on both the substrates [62]. Thicker Al_2_O_3_ nanocoatings (50 nm thickness) produced by both ALD techniques showed better resistance to corrosion due to their low porosity and strong adherence to substrates [62]. The presence of porosity and weak adhesion of nanocoatings to substrates are detrimental to corrosion resistance. Overall, it was concluded that nanocoatings deposited by plasma-enhanced ALD provide higher corrosion resistance, and that 50 nm thick nanocoatings produced by both ALD techniques provide the best corrosion resistance. A preliminary step that can improve the corrosion resistance of nanocoatings is their pre-treatment. Hydrogen–argon plasma pre-treatment of Al_2_O_3_ nanocoatings deposited on steel via both plasma-enhanced ALD and thermal-enhanced ALD and its effect on corrosion performance of the deposited nanocoatings demonstrated this fact [63]. Plasma pre-treatment and increased pre-treatment time improve corrosion resistance in the nanocoatings produced by both methods. The improvement was observed to be more pronounced in thermal-enhanced ALD coatings due to their enhanced adhesion to substrates and reduced porosity, imparted by the pre-treatment. Surface treatments such as pre-annealing of substrates before the deposition of nanocoatings results in the removal of heterogeneities, resulting in better formation of nanocoatings. Pre-annealing of copper substrates and subsequent deposition of 10–50 nm Al_2_O_3_ coatings by ALD [64] has shown enhancement in the corrosion resistance of the nanocoatings. In the ALD process, it has been observed that the deposition temperature influences the corrosion behaviour of nanoceramic coatings. The corrosion resistance of Al_2_O_3_ nanocoating deposited on 316 L stainless steel with ALD at the temperature of 250 °C was found to be superior to that of the coating deposited at 160 °C. Deposition at higher temperature improves the coating’s sealing effectiveness, i.e., reduces porosity and thereby improves corrosion resistance [65]. Certain carbon steels, on the other hand, require lower deposition temperatures to avoid adverse effects on their microstructure. Al_2_O_3_ nanocoating deposited on 100Cr6 carbon steel at 160 °C using the ALD process requires a nanocoating thickness of >10 nm to achieve effective sealing and avoid corrosion [66]. In view of the above discussion, it can be concluded that the synthesis parameters have a vital role in determining the properties of the nanocoating.

#### 3.1.2. Titanium Oxide (TiO_2_) Nanostructured Coatings

Titanium oxide (TiO_2_) is a popular ceramic material known for its resistance to corrosion and mechanical abrasion [67], photocatalysis [68], protection against UV [69] and self-cleaning property [70].

**Figure 3:**
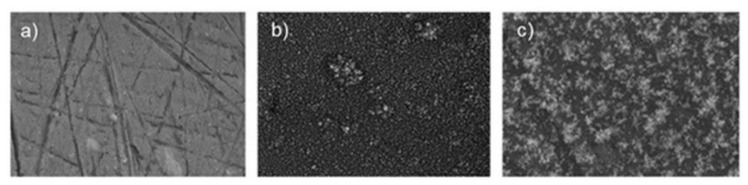
SEM images of (**a**) uncoated AA2024 sample, (**b**) TiO_2_-coated AA2024 with deposition time of 40 s and (**c**) TiO_2_-coated AA2024 with deposition time of 80 s [73].

A factor that controls the corrosion resistance of ceramic nanocoatings is the size of nanoceramic particles. Corrosion studies of TiO_2_ nanocoating on carbon steel substrate showed improved corrosion resistance with the reduction in the size of nano-TiO_2_ particles [71]. Corrosion rates of nanocoatings with 10 nm, 50 nm, 100 nm and 150 nm TiO_2_ particle sizes in 1 M H_2_SO_4_ solution (determined using polarisation and electrochemical impedance spectroscopy), deposited on carbon steel surfaces, revealed that these nanocoatings prevent corrosion. However, it was found that the physical adhesion of the nanocoatings to the substrate surface depends on the nanoparticle size—the smaller the particle size, the better the coating–substrate interface bonding. Corrosion resistance of nanocoatings improved with the reduction in the size of nano-TiO_2_ particles primarily due to decreased O_2_ and H_2_O permeability into nanocoatings [71]. The addition of graphene oxide (GO) to TiO_2_ nanocoatings has shown a significant improvement in the anticorrosion performance of the nanocoatings. Nanocomposite TiO_2_/GO (graphene oxide) ceramic coating produced for a cast iron pipeline showed a remarkable 94% reduction in corrosion rates compared to bare substrate in seawater. This is mainly due to the reduced porosity and capacitance of the coating [72]. Another factor that controls corrosion behaviour of TiO_2_ nanocoatings is their thickness. Figure 3 shows scanning electron microscope (SEM) images of an uncoated AA2024 aluminium alloy substrate, and a substrate with TiO_2_ nanocoatings deposited at time durations of 40 s and 80 s [73]. The TiO_2_ nanocoating with greater thickness achieved by longer deposition time had the best corrosion resistance, with current density lower by one order of magnitude.

#### 3.1.3. Tantalum Pentoxide (Ta_2_O_5_) Nanostructured Coatings

Tantalum pentoxide (Ta_2_O_5_) ceramic is an excellent material for making corrosion-resistant nanocoatings [74]. Ta_2_O_5_ has high hardness [75] and high resistance to chemical attack under extreme environments [76]. Due to its high dielectric constant (~25), it is used in capacitors for automobile electronics, high-speed tools and cell phones [77,78,79]. 

β-Ta_2_O_5_ nanoceramic nanocoating on a Ti-6Al-4V alloy substrate enhanced corrosion resistance in 3.5 wt % NaCl due to the formation of a stable passive oxide layer [80]. A comparative corrosion performance of filtered cathodic arc deposited (FCAD) tantalum oxide (Ta_2_O_5_) and chromium oxide (Cr_2_O_3_) nanocoatings on 100Cr6 steel substrate showed that the substrate coated with Ta_2_O_5_ nanocoating exhibited improved corrosion resistance than that coated with Cr_2_O_3_ nanocoating [76]. The deposition method also influenced corrosion performance, such that FCAD Ta_2_O_5_ nanocoatings exhibited nearly four times higher corrosion resistance than that of the nanocoatings deposited by ALD. The spurious interfacial oxide layer generated in ALD coating increases voids at the interface, coating degradation and dissolution of the coating. In the FCAD process, however, the native oxide layer is removed by pre-etching substrate surfaces by ion bombardment prior to actual oxide growth (passive oxide coating) [81].

#### 3.1.4. Tantalum Nitride (Ta_2_N) Nanostructured Coatings

**Figure 4:**
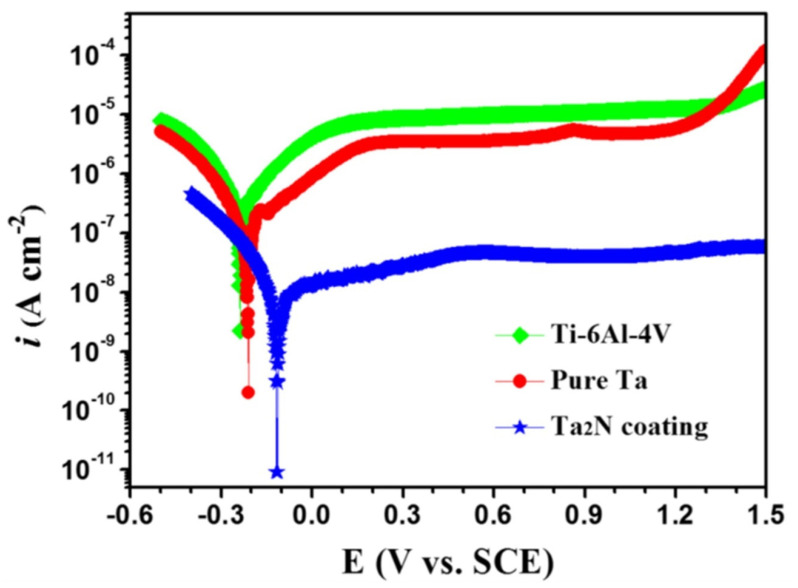
Potentiodynamic polarisation curves of bare Ti-6Al-4V, pure Ta and Ta_2_N coating [83].

Tantalum nitride (Ta_2_N) deposited over Ti-6Al-4V bipolar plates using the reactive sputter deposition method [82] was studied for its corrosion properties in the simulated polymer electrolyte membrane fuel cell environment, with varying pH values and temperatures. The Ta_2_N nanocoated substrate had significantly higher corrosion resistance when compared to the uncoated Ti-6Al-4V at any particular pH or temperature value. An increase in acidity showed a reduction in corrosion resistance [82]. Investigation of nanocoatings on Ti-6Al-4V for biomaterial applications has shown that Ta_2_N nanocoating exhibited better corrosion resistance in Ringer’s physiological solution at 37 °C with lower Icorr values when compared to both pure Ta and bare Ti-6Al-4V, as can be seen from Figure 4 [83]. 

Tantalum-based nanocoatings of Ta_2_O_5_, Ta_3_N_5_ and TaON (tantalum oxynitride) deposited on 306 stainless steel [84] showed enhancement in their corrosion performance by impeding the corrosion current density due to the formation of a passive film. Corrosion rates reduced by almost 50% when compared to the bare stainless steel and pure tantalum-coated stainless steel samples. TaON (tantalum oxynitride) demonstrated the best corrosion resistance among the Ta-based nanocoatings, followed by Ta_2_O_5_ (tantalum pentoxide) and Ta_3_O_5_ (tantalum nitride). The better corrosion resistance of TaON was attributed to its hydrophobic nature, aided by its texture. Furthermore, it was observed that the anticorrosive nature of the TaON nanocoating was influenced by the morphological, chemical and electrical properties of the deposited film. Thus, the TaON nanocoating significantly reduced the corrosion current density, resulting in enhanced anticorrosive behaviour [84]. 

### 3.2. Metallic Nanostructured Coatings

Different factors influence the corrosion behaviour of metallic nanocoatings, such as grain size, composition of nanocoating, synthesis method, operating environment and incorporation of additives [11]. The effects of each of these factors on corrosion behaviour of metallic nanocoatings are discussed in this section.

#### 3.2.1. Grain Size

Grain size can significantly affect the corrosion performance of nanocoatings. Ni nanocoatings with 50 nm grain size were deposited on Q235 steel by pulse electrodeposition [85]. The nanocoatings were annealed in vacuum (200 °C and 400 °C; 10 min). After annealing, it was observed that the grain size increased to 60 nm when annealed at 200 °C, and to 500 nm when annealed at 400 °C. The corrosion resistance of the nanocoatings was evaluated in solution of 0.1 mol/L H_3_BO_3_ + 0.025 mol/L Na_2_B_4_O_7_ + 0.02 mol/L NaCl (pH 8.4). It was found that the annealed coatings having larger grain size showed better resistance to corrosion. Of note, the usual grain size effect on corrosion behaviour was not observed in this case. 

Such a behaviour was attributed to the formation of twins during annealing. It was observed that the density of twins increased with increase in grain size. Twin boundaries possess low free energy, which promotes corrosion resistance. Hence, upon annealing, increased twin density enhanced the homogeneity of the passive film, thereby reducing the susceptibility of the annealed coatings to pitting corrosion, even though the annealed coatings had larger grain size [85].

Corrosion studies of nanocrystalline nickel–tungsten (Ni-W) nanocoatings [86] have shown that (i) the corrosion rate decreases with increasing grain size in alkaline solution (pH 10) and that (ii) the corrosion rate increases with the increase in grain size in acidic solution (pH 3), indicating that the pH also influences the corrosion behaviour of the nanocoatings along with their grain size. 

This difference in behaviour of Ni-W nanocoatings (grain size: 5 to 63 nm) in solutions of varying pH values was observed to be influenced by two competing factors [86]: (i) the formation of passive oxide film, which depends on W content, and (ii) active sites available for corrosion to occur, which depends on grain boundary volume. Hence, in alkaline solution, wherein corrosion increased with the decrease in grain size, the occurrence of corrosion was ascribed to the increased grain boundary volume. In contrast, in an acidic saline environment, the corrosion rate decreased with the decrease in grain size. In this case, corrosion was dominated by W content, such that coatings with higher W content showed better corrosion resistance due to strong oxide film formation. Nanocoatings in alkaline solution showed better corrosion resistance than in acidic solution [86].

The corrosion resistance of nanocrystalline nickel nanocoatings produced on steel substrates by reverse pulse electrodeposition method was improved with a reduction in grain size, in 10% HCl and a neutral spray solution test [87]. pH value had a considerable effect on the grain size and consequently on the corrosion behaviour of pure cobalt nanocrystalline nanocoatings electrodeposited on stainless steel substrate [88]. Corrosion tests conducted for the nanocoatings deposited at pH of 3, 5 and 7 showed that the best corrosion resistance was obtained for smaller grain size nanocoatings, when the coating was electrodeposited at a pH of 3 [88]. Grain size effect on corrosion rate has also been observed in Ni-Gr, Ni-ZrO_2_ and Ni-Al_2_O_3_ nanocomposite coatings. The corrosion rate increases with the increase in grain size due to increased porosity, stresses at the surface and permeability [89]. 

#### 3.2.2. Composition

The incorporation of metallic reinforcements in nanocoatings can greatly improve corrosion properties of metallic nanocoatings. When compared to microscale reinforcements, nanocrystalline reinforcements prevent clustering of reinforcements [90]. The corrosion resistance of pure metallic nanocoatings is usually not sufficient in harsh conditions, and hence, they are alloyed with other metals to improve their corrosion resistance [11].

Studies [91,92] have shown that the content of Ni in alloyed Zn-Ni nanocoatings, electrodeposited on carbon steel, is a key factor that determines the response of the nanocoatings to corrosion. In the range of Ni content from 0% to 19.54%, the best corrosion resistance was achieved for 13 wt % with 26 nm grain size, [91] and in the range of Ni content from 12% to 18%, the best corrosion resistance was achieved for 17.62 wt % of Ni with 37 nm grain size [92]. Similarly, the content of Mo in the Ni-Mo alloy nanocoatings, synthesised by electrodeposition, was critical in influencing their corrosion behaviour [93]. These coatings, when tested for their corrosion in H_2_SO_4_ solution, showed that with the increase in Mo content, the corrosion resistance of the nanocoatings increased. The best corrosion resistance was observed for the nanocoatings having Mo 16.7 at %. However, as the Mo content further increased, corrosion resistance declined due to pitting corrosion [93]. Similarly, the best corrosion resistance for nano-Ni-Mo alloy nanocoatings in 0.5% NaCl solution occurred at the 19 wt % of Mo content, in the range of Mo content from 11% to 32% [94]. Enhancement in the corrosion resistance of nano-Ni-Mo nanocoatings was due to refinement in grains, i.e., reduction in grain size due to increased Mo content in Ni-Mo nanocoatings [95]. When the effect of cobalt concentration on the corrosion performance of Ni-Co nanocrystalline nanocoatings with 0–45 wt % Co was evaluated [96], the Ni-17 wt % Co alloy showed better corrosion performance compared to nanocoatings with 0, 8, 24, 32, 38, 42 and 45 wt % of cobalt [96]. The higher corrosion resistance of Ni–17 wt% Co alloy coating was attributed to its microstructural features, such as (i) the formation of single-phase f.c.c. structure, (ii) moderate grain size (~50 nm) and (iii) predominance of the close-packed (111) preferred orientation [96]. The corrosion resistance of metallic nanocrystalline coatings can be improved by adding selective second-phase particles. However, some works report decreased corrosion resistance with particle addition, such as the inclusion of SiO_2_ and diamond in Ni-W and Ni-Mo nanocoatings, respectively [97,98].

#### 3.2.3. Synthesis Method

**Figure 5:**
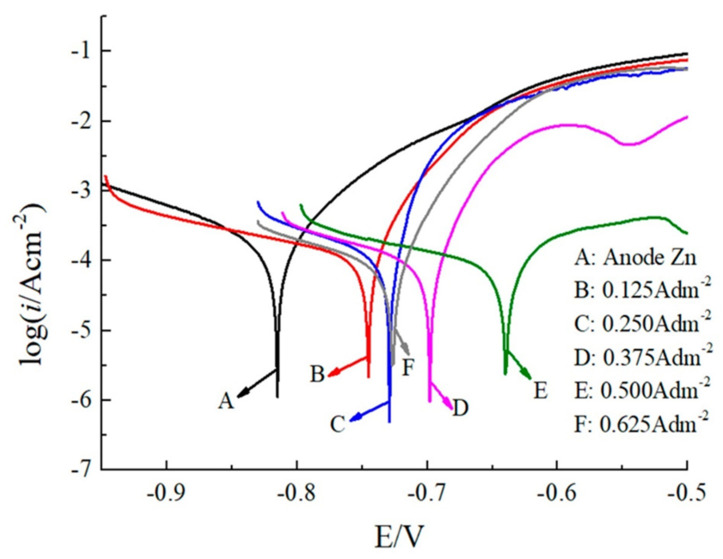
Tafel curves of deposited Zn coating at varying current densities [102].

The synthesising method influences the corrosion properties of metallic nanocoatings. Electrodeposition is the most commonly used method for synthesising metallic nanocoatings. Direct currents or pulsed currents can be used for electrodeposition. Cobalt–phosphorous (Co-P) nanocoatings deposited on mild steel substrate through pulse electrodeposition exhibited a lower corrosion rate compared to nanocoatings deposited through direct current (DC) electrodeposition in non-deaerated NaCl solution [99]. When compared to direct current electrodeposition, zinc nanocrystalline coatings deposited on steel substrates through pulse electrodeposition were found to be less porous, and hence more corrosion-resistant [100,101]. It has been reported that the corrosion resistance of nanocrystalline zinc deposited on copper substrate at varying current densities showed significant variation [102]. When the current density at which the nanocrystalline zinc nanocoating was deposited increased from 0 to 0.5 A/dm2, both the Icorr value and the corrosion rate reduced significantly; however, when the current density increased further to 0.625 A/dm^2^, the corrosion rate increased again. The nanocoating deposited at a current density of 0.5 A/dm^2^ exhibited the best corrosion resistance [102]. This is due to the fact that smaller grain size and uniform dispersion were achieved at 0.5 A/dm^2^ compared to that at other current densities. Tafel polarisation curves of these nanocoatings are shown in Figure 5.

#### 3.2.4. Environment

pH of the corrosive fluid showed a significant influence on the corrosion resistance of Ni-W nanocoatings [86]. Corrosion behaviours of nanocrystalline nickel–tungsten nanocoatings in both alkaline solution of 3.5 wt % NaCl with pH of 10 and an acidic solution of 3.5 wt % NaCl with pH of 3 were evaluated [86]. Corrosion rates were higher in the acidic solution (pH 3) compared to the alkaline solution (pH 10) [86]. When nanocrystalline cobalt coatings were evaluated for their corrosion performance at three different pH levels of 3, 5 and 7, the Co nanocoatings tested at pH 3 performed better than the coatings tested at pH 5 and 7 [88], highlighting that Co nanocoatings are highly resistant to corrosion in acidic solutions. This is due to the agglomeration of grains at higher pH values. As the grain size at acidic pH of 3 was smaller, the results showed better corrosion resistance [88]. 

In sulphur dioxide (SO_2_) environment, prevalent in some industries, nickel coatings containing graphene (Ni-Gr) have shown better corrosion resistance than nickel coatings having alumina (Ni-Al_2_O_3_) and zirconia (Ni-ZrO_2_) [103].

#### 3.2.5. Additives

The inclusion of additives has a beneficial effect on the corrosion behaviour of nanocoatings. As an example, a nickel–tungsten (Ni-W) nanocrystalline coating electrodeposited from a citrate bath having salicylaldehyde additives in concentrations ranging from 0–500 ppm was examined [104]. Corrosion resistance was the best at the salicylaldehyde concentration of 100 ppm, as the coating generated was homogeneous, smooth and fine-grained, resulting in increased corrosion resistance. Concentrations > 100 ppm resulted in decreased corrosion resistance [104]. The addition of saccharin to the plating solution to deposit nanocrystalline nickel nanocoatings lowered the corrosion rate by reducing the grain size of the nanocrystalline nickel [87], such that at 5 g/L saccharin concentration, the average grain size reduced from 32.40 nm to 13.05 nm. Saccharin addition giving rise to increased corrosion resistance has also been reported for nanocrystalline Ni nanocoatings (formed on Q235 steel substrate by the pulse jet electrodeposition technique) [85]. 

The addition of phytic acid at concentrations of 0.1, 0.2, and 0.3 g/L to nanocrystalline nickel coating has shown variation in corrosion resistance of the coatings. Nanocoatings produced from 0.2 g/L phytic acid solution showed the best corrosion resistance. Such an occurrence was attributed to the fine and homogenous microstructure and morphology obtained at this concentration [105]. The corrosion resistance of nanocrystalline nickel coated on glass substrates using DC magnetron sputtering was evaluated to examine the influence of Cr addition and its concentration on the corrosion resistance of the nanocoating. Potentiodynamic tests revealed that the corrosion resistance of the Ni nanocoating increased with the increase in Cr concentrations up to 25 wt %. Ni nanocoatings containing 25 wt % Cr showed the best corrosion performance [106]. 

**Figure 6:**
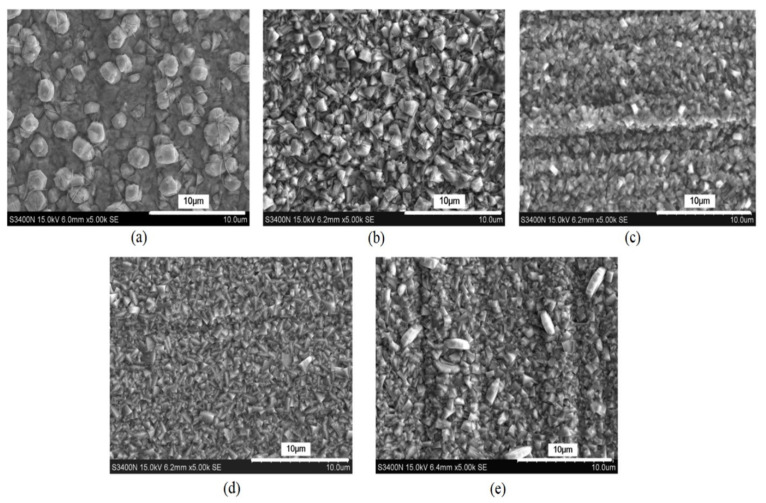
Surface morphology of nanocoating at varying concentrations of CPAM (**a**) 5, (**b**) 10, (**c**) 15, (**d**) 20, and (**e**) 25 g/L [102].

**Figure 7:**
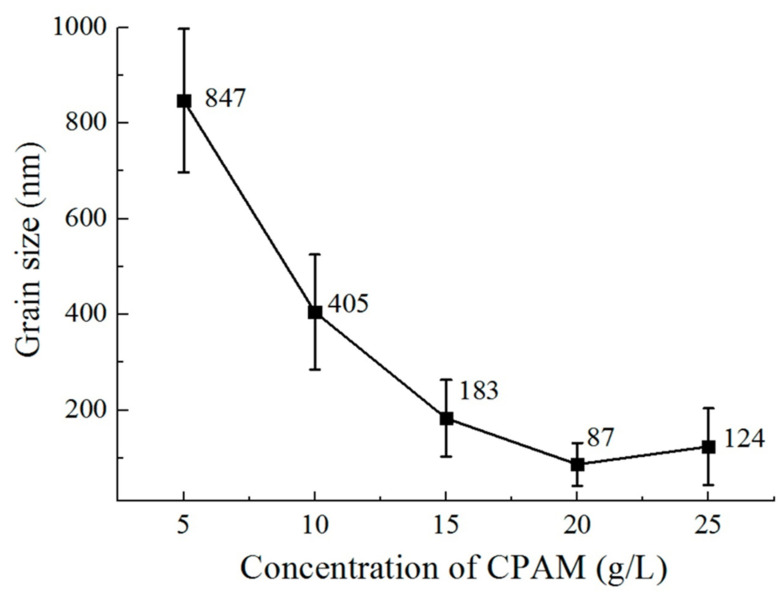
Variation in grain size in nanocrystalline zinc coatings with concentration of CPAM [102].

Nanocrystalline zinc deposited using ZnSO_4_ electrolyte with cationic polyacrylamide (CPAM) as a polymeric additive showed a reduction in grain size when the concentration of CPAM was increased from 5 g/L to 20 g/L. At 20 g/L concentration of CPAM, grains were found to be refined, which resulted in better corrosion resistance [102]. However, further increase in the concentration of CPAM to 25 g/L resulted in large grain size and loss of uniform distribution of grains [102]. Figure 6 shows the surface morphology of the coatings with varying concentrations of CPAM. Figure 7 shows the variation in grain size as a function of concentration of CPAM [102].

### 3.3. Nanocomposite Coatings

The development of nanocomposite coatings is a rapidly growing field in the domain of nanotechnology. Nanocomposite coatings are rapidly being inducted in the sectors of aerospace [107,108], marine [109], automobiles [110], sensors [111], dental implants [112,113] and electronics [114]. Factors that affect the functionality of nanocomposite coatings include properties of matrices and fillers, spatial dispersion of fillers, surface morphology and deposition techniques [108,115]. The corrosion behaviour of nanocomposites with polymer/metal matrices is presented in this section.

#### 3.3.1. Polymer Matrix Nanocomposite Coatings

Polymer nanocomposite coatings which use polymers as matrices have received considerable interest in anticorrosion applications. By incorporating nanomaterial fillers in polymer matrices, improvement in several properties can be achieved, such as, stiffness, strength, corrosion resistance and wear resistance [116,117,118].

Nanostructured chitosan/ZnO coating was found to suppress corrosion on mild steel, with corrosion resistance improving as a function of increasing the number of layers of chitosan/ZnO [119]. Nanocomposite coating of oleic acid-modified chitosan/graphene oxide layer (CS/GO-OA) on a carbon steel substrate in NaCl solution increased corrosion resistance by 100-fold [116]. This improvement in corrosion resistance was ascribed to the decrease in hydrophilicity, oxygen permeability and ion transport because of the presence of the nanocomposite coating. Hydrophilicity of the nanocomposite coating was lowered because of the presence of a large alkyl group of oleic acid, whereas the formation of a barrier on the coating due to the interaction of functional groups between chitosan and oleic acid reduced ion transport through the nanocomposite coating. Graphene oxide reduced oxygen permeability [116].

The influence of graphene nanoplatelets (GNPs) on corrosion resistance of UHMWPE/GNPs nanocoatings deposited on AA2028 aluminium alloy substrate by electrostatic spraying was evaluated by comparing its corrosion resistance with that of the uncoated substrate and pure coating (pristine UHMWPE) [120]. Nanocoating with 2 wt % GNPs showed the maximum corrosion resistance, in 3.5% NaCl solution [120]. Mild steel coated with polyaniline coatings [121] containing 0% graphene (Pani) and PaniGn coatings containing 0.49, 1.92, 8.91 and 16.37 wt % graphene exhibited a significant corrosion reduction, by about 3–4 orders of magnitude, as compared to the uncoated mild steel. The nanocomposite coatings served as a physical barrier to the corrosive HCl environment while simultaneously imparting non-wetting properties. The coating with 1.92 wt % graphene provided the best corrosion resistance. Electrodeposited PaniGn nanocomposite coatings also improved the corrosion resistance of copper in 5000 ppm NaCl. The graphene-reinforced polyaniline coating generated a dense and compact layer, resulting in lower values of metal substrate corrosion potential and a lower rate of corrosion [122]. 

Anticorrosion performance can be greatly enhanced by incorporating treated nanoparticles in nanocomposite coatings. As an example, silicon dioxide (SiO_2_) nanoparticles were surface-treated with poly (styrene-co-butyl acrylate) to improve their dispersion in a fluoropolymer coating [123]. Enhanced corrosion resistance of the fluoropolymer nanocomposite coatings with treated silica nanoparticles up to a 4 wt % concentration of SiO_2_ was observed when coated on a steel substrate, compared to that of the uncoated steel substrate. However, the addition of SiO_2_ > 4 wt % weakened the link between the nanocomposite coating and the substrate, causing the nanoparticles to agglomerate, resulting in lower corrosion resistance [123].

#### 3.3.2. Waterborne Polymer Nanocomposite Coatings

Volatile organic compounds (VOCs) are often used as plasticisers in paints to facilitate polymer dispersion and reduce ductility. However, the use of such substances is extremely detrimental to the environment [124,125]. A waterborne polymer coating, which uses water as a solvent instead of VOCs, was developed [126]. In comparison to the health risks and toxicity issues created by VOCs [125], waterborne polymer coatings provide advantages such as eco-friendliness, low viscosity, ease of cleaning, and non-toxicity [124]. Several researchers have investigated the corrosion behaviour of polymer-based waterborne coatings embedded with nanoparticles, such as Fe_3_O_4_**,** Fe_2_O_3_ and ZnO. 

**Figure 8:**
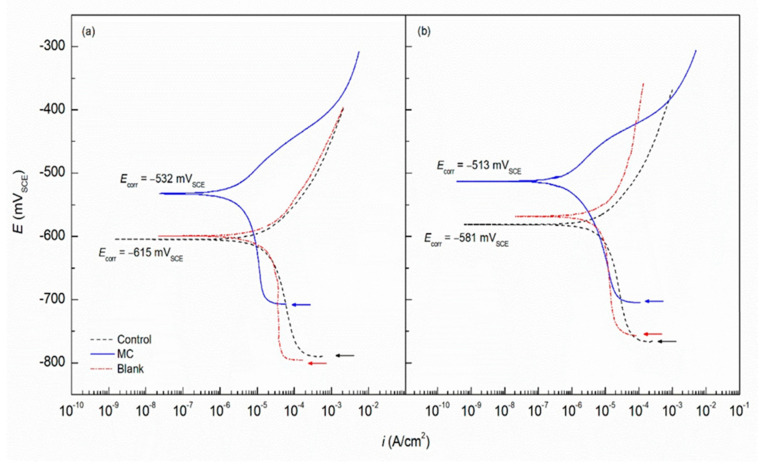
Potentiodynamic curves with acrylic coating doped with colophony microcapsules deposited on mild steel in (**a**) DI water (pH 6.8) and (**b**) SCPS (pH 12.6) [127].

**Figure 9:**
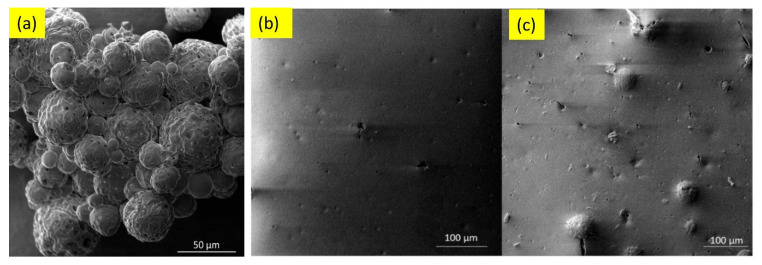
SEM images of (**a**) colophony microcapsules, (**b**) acrylic coating without microcapsules and (**c**) acrylic coating doped with microcapsules [127].

Waterborne epoxy acrylate-butylated melamine formaldehyde (EpAc-BMF) and ferrite (Fe_3_O_4_) nanocomposite coatings were examined for their corrosion performance (EpAc-BMF-Fe_3_O_4_) [124]. Corrosion resistance was tested in NaOH, NaCl and HCl solutions. EpAc-BMF-Fe_3_O_4_ nanocomposite coatings increased the corrosion resistance of mild steel samples in a salt spray test. An epoxy-based coating creates a protective barrier that prevents corrosive and aggressive ions from penetrating the steel surface [124]. The corrosion protection effect of colophony microcapsules incorporated in a waterborne acrylic coating, coated on a carbon steel substrate was examined [127]. It was observed that the addition of microcapsules improved corrosion resistance of the waterborne coatings. In two separate solutions with varying pH values, the microcapsule-doped coating maintained more noble Ecorr values and lower corrosion current density (Figure 8). Figure 9 shows SEM images of colophony microcapsules and coated steel specimens with and without doped microcapsules [127].

#### 3.3.3. Metallic Matrix Nanocomposite Coatings

Reinforcements such as ceramic nanoparticles and carbon nanotubes that are inherently resistant to corrosion are incorporated in metallic matrices to produce nanocomposite coatings with increased corrosion resistance. Some examples are given here. The incorporation of SiC nanoparticles in Ni and Ni alloys resulted in the enhancement of corrosion resistance of the nanocomposite coatings [128,129]. Ni-P electroless coatings incorporated with SiC, Al_2_O_3_ and CeO_2_ nanoparticles increased their anticorrosion ability in NaCl and H_2_SO_4_ solutions. The addition of nanoparticles of SiO_2_ [130], Al_2_O_3_ [131,132,133] and CeO_2_ [134] to Ni-P electroless coatings improved the corrosion resistance of nanocomposite coatings in NaCl and H_2_SO_4_ solutions. The addition of carbon nanotubes (CNT) showed increased corrosion resistance of electroless Ni-P-CNT nanocomposite coatings in NaCl solution [135]. 

When compared to pure Zn coating, electrodeposited Zn-TiO_2_ nanocomposite coating performed better in (NH_4_)_2_SO_4_ solution with pH of 3 [136]. The best corrosion resistance was observed at 5 g/L nano-TiO_2_ concentration, due to inert oxide particles reducing the active surface in contact with the corrosive environment. The deterioration of corrosion performance at 10 g/L nanoparticle concentration was attributed to TiO_2_ nanoparticle aggregation and their non-uniform distribution [136]. 

## 4. Tribological Performance

Tribological issues, namely friction and wear, manifest on surfaces that undergo relative mechanical motion, such as in gears, bearing, motor shafts, etc. About 23% of the energy consumption in the four main energy-consuming sectors—transportation, manufacturing, power generation and residential—is due to tribological issues, of which 20% is to overcome friction [137]. According to a recent report from the United States Department of Energy, new technologies that can be achieved via targeted research projects in tribology could save up to 2.1% of the GNP of energy annually [138]. In this context, the development of tribologically beneficial nanocoatings has gained importance. With the right selection of nanomaterials, (i) coatings with high hardness and high fracture toughness can be realised to enhance the wear resistance of surfaces, and (ii) coatings with solid lubricants can be realised to reduce friction effectively at sliding interfaces. The tribological behaviours of various nanocoatings are discussed in this section.

### 4.1. Ceramic Nanostructured Coatings

Ceramic nanocoatings exhibit superior wear resistance due to their small grain size, which provide enhanced toughness [139]. Ceramic nanocoatings are being used in fields such as dentistry, bio-implants [140,141,142,143], etc.

#### 4.1.1. Zirconia (ZrO_2_)-Based Nanostructured Coatings

Zirconia (ZrO_2_) nanocoatings exhibit superior tribological properties and are also attractive for their electrical and optical properties [144,145,146,147,148,149]

Nano-ZrO_2_ films coatings were deposited on 304 stainless steel substrates [150]. The nanofilms exhibited lower friction coefficients when slid against a SiC grinding ball in 5% NaCl solution, distilled water and in dry conditions. While adhesive and oxidation wear were the dominant wear mechanisms under dry conditions, in 5% NaCl solution, it was corrosive wear [150]. When compared to micro-sized zirconia coatings, nanostructured ZrO_2_-3 mol % Y_2_O_3_ nanocoatings deposited by air plasma spray showed a lower wear rate [151]. In simulated body fluid conditions, a bilayered nanocoating (ZrO_2_/Al_2_O_3_-13TiO_2_) coated on a Ti-13Nb-13Zr alloy showed improved wear resistance when compared to both ZrO_2_ and Al_2_O_3_-13TiO_2_ nanocoatings. This enhancement was due to lower porosity and increased adhesive strength of the bilayered coatings [141]. 

Yttria stabilised zirconia (YSZ) ceramics have piqued the interest of many researchers and have been widely used in several applications [152,153,154]. In YSZ, yttrium oxide stabilises the cubic crystal structure of zirconium dioxide at ambient temperature. Tribological and mechanical properties of YSZ nanocoatings coated on Ni superalloy vary with the plasma current used in the air plasma spraying process. Plasma current has been found to impact the percentage of nano zones, which in turn influence the wear rate of the nanocoatings. As the wear rate is influenced by particle agglomeration in the coating, a higher temperature achieved by a higher plasma current triggers the melting of particle agglomerates. This melting improves the nanocoating by enhancing its mechanical integrity and thus its wear rate is lowered. The nanocoatings outperformed micro-sized YSZ coatings in terms of wear resistance [155]. Other research works [156,157] on tribological performance of YSZ have also shown that nanostructured coatings exhibited better wear resistance compared to microcoatings.

#### 4.1.2. Alumina (Al_2_O_3_)-Based Nanostructured Coatings

Alumina (Al_2_O_3_), an oxide ceramic, has been widely used as a coating material as it has excellent wear resistance [23]. The wear behaviour of nanostructured alumina coatings coated on SS304 stainless steel substrates by atmospheric plasma spraying was evaluated and compared to that of micro-alumina coatings [158]. At the applied normal loads ranging from 30 N to 80 N, nano-alumina coatings outperformed microcoatings in terms of wear resistance [158]. Similar results have been observed for nano-Al_2_O_3_ coatings on SS304 substrate [159]. Atmospheric plasma-sprayed nanostructured alumina-titania ceramic coating in comparison to micro-sized alumina-titania coatings has shown lower material loss and less friction [160]. The better tribological performance of the nanocoatings has been ascribed to a bimodal (completely melted and unmelted or partially melted) microstructure and higher hardness of the nanoceramic coating [160]. An investigation on the tribological behaviour of Al_2_O_3_-13 wt % TiO_2_ nanoceramic coating and ZrO_2_ nanoceramic coating, both thermally sprayed on pure titanium and titanium alloy (Ti–13Nb–13Zr) substrates, was conducted. When tested against alumina balls, the wear resistance of the alumina-titania (Al_2_O_3_-13 wt % TiO_2_) coating was superior (i.e., better wear resistance) when compared to the nano-zirconia (ZrO_2_) coating. The higher wear resistance of alumina-titania nanocoatings was attributed to its higher toughness due to the addition of TiO_2_ particles [161]. Nanostructured Al_2_O_3_-13 wt % TiO_2_ coatings deposited on SAE-1042 steel by atmospheric plasma spraying showed improved wear resistance compared to microcoatings [162]. The enhancement in wear resistance of the nanostructured coating was attributed to its composite hierarchical microstructure, which facilitated crack deflection that acted as a toughening mechanism [162].

#### 4.1.3. Chromia (Cr_2_O_3_)-Based Nanostructured Coatings

Chromium oxide (Cr_2_O_3_) coatings have high wear resistance. Due to their high melting point, chromium oxide coatings are deposited using the plasma spraying process, which is a high temperature coating deposition technique [163,164,165,166]. When compared to micro-sized Cr2O_3_ coatings, plasma-sprayed nanostructured Cr_2_O_3_ coatings deposited on stainless steel and SS304 substrates showed higher wear resistance [167,168]. Thermal-sprayed nanostructured Cr_2_O_3_ nanocoatings have also shown higher wear resistance compared to micro-sized Cr_2_O_3_ coatings [169]. The effect of the addition of YSZ (Yttria-Stabilised Zirconia) and SiC (silicon carbide) reinforcements to pure Cr_2_O_3_ ceramic coatings deposited by plasma spraying on 304 L stainless steel substrates was studied [170]. Wear behaviour of Cr_2_O_3_, Cr_2_O_3_-20YSZ and Cr_2_O_3_-20YSZ-10SiC coatings showed that YSZ-reinforced nanocoatings (Cr_2_O_3_-20YSZ) outperformed pure Cr_2_O_3_ and Cr_2_O_3_-20YSZ-10SiC coatings in terms of wear resistance. The improved wear resistance of Cr_2_O_3_-20YSZ was attributed to its phase transformation toughening mechanism. This is due to the presence of ZrO_2_ (tetragonal) in the Cr_2_O_3_-20YSZ coating and higher plastic deformation and tribo-film formation during wear [170]. The tribological behaviour of nanostructured Cr_2_O_3_-3% TiO_2_ was compared to that of a micro-sized Cr_2_O_3_-3% TiO_2_ coating deposited on stainless steel substrates [171]. When compared to the microcoatings, nanostructured coatings showed a lower friction coefficient and lower wear rate. Changes in the microstructure of the nanocoatings improved their tribological performance. The nanocoatings consist of partially melted regions and exhibited a bimodal microstructure, whereas in the conventional coating, fully melted splats with some porosity were observed. This microstructural difference between the micro- and nanocoatings was attributed to the powders used. Conventional powders (i.e., micron-sized powders) resulted in higher splashing because of their larger size, which in turn leads to reduced adhesion of the coatings to their substrates [171].

#### 4.1.4. Other Ceramic Nanostructured Coatings

Tantalum pentoxide (β-Ta_2_O_5_) nanoceramic coating deposited on Ti-6Al-4V alloy substrate, produced using a double glow discharge plasma technique, showed an enhancement in wear resistance over the bare alloy substrate by two orders of magnitude. The higher wear resistance is due to the β-Ta_2_O_5_ coating’s good mechanical characteristics combined with its high adhesion [80]. When Ti-6Al-4V alloys were coated with two different types of nanoceramic coatings, namely β-Ta_2_O_5_ and TaON [172], and studied for a dry sliding wear test using a ball-on-disk tribometer against Si3N4 balls (applied loads: 2.3 to 5.3 N), both the coatings showed a reduction in their specific wear rate by two orders of magnitude when compared to the uncoated Ti-6Al-4V alloy substrate. The lowering of the wear rate is caused by the reduced real contact area and higher surface hardness [172]. 

### 4.2. Metallic Nanostructured Coatings

Metallic nanocoatings can be of a single metal, such as iron, zinc or tungsten, or they can be alloyed to improve coating properties. The addition of metal to a nanocoating improves its physical and mechanical properties [173,174,175]. The influence of factors such as coating method, composition of nanocoatings and grain size of nanocoatings on tribological performance of metallic nanocoatings is discussed in this section.

#### 4.2.1. Coating Method

Nanocoating prepared from mechanically alloyed NiAl powder was deposited on low-carbon steel substrate via the high-velocity oxygen fuel (HVOF) thermal spray technique. Wear behaviour (applied normal loads: 30, 60 and 90 N) of NiAl nanocoating at two distinct fuel/oxygen ratios showed that nanocoatings produced at higher fuel/oxygen ratios exhibited higher mass loss, i.e., higher wear. Higher Al oxidation occurs in coating produced at a higher/fuel oxygen ratio, which results in lower hardness and consequently higher wear [176]. When wear and friction behaviour of NiAl coatings generated by laser cladding at varying laser power densities were examined [177], it was observed that the laser power density influenced their tribological performance. Apart from having low coefficient of friction and wear rate, the tribological performance was significantly influenced by the contact load and sliding speed for the NiAl coatings developed at low power densities [177]. 

In the electrodeposition of metallic nanocoatings, the applied current density during deposition influences the properties of nanocoatings [178]. Fe-Ni alloy nanocoating prepared by pulse electrodeposition at various current densities (10, 20, 30, 40 and 50 mA/cm^2^) deposited on copper was evaluated for its sliding wear behaviour (applied normal load: 3 N). The results showed that nanocoatings deposited at higher current densities experienced lower friction coefficient and lower wear rates. This was due to the decreased Fe content and smaller grain size obtained at higher current densities. Furthermore, as iron is softer than nickel, wear was higher in coatings with high iron content [178]. The wear rate of nanocrystalline Co-W alloy coating generated by dual pulse electrodeposition, deposited on copper [179], decreased with the increase in current density up to the current density of 4 A/dm^2^. However, as the current density increased further (>4 A/dm^2^), the wear rate increased, and the frictional coefficient decreased. This is due to the rougher surface and aggregating crystal boundaries in coatings produced at higher current densities [179]. Nanocrystalline zinc coating produced with the pulse reverse current electrodeposition method, deposited on steel, showed lower wear rate when compared to direct current and pulse current electrodeposition techniques. The lower wear rate in coatings produced by pulse reverse current was attributed to the enhanced hardness in coatings [180]. Another coating process parameter that influences the tribological behaviour of metallic nanocoatings is the substrate temperature. In DC magnetron sputtering, the substrate temperature is critical for the properties of the nanocoating. As an example, nanocrystalline Cu coatings generated by DC magnetron sputtering on silicon substrates at different substrate temperatures (ambient temperature, 100 °C and 200 °C), showed that the coatings deposited at 100 °C had reduced values of frictional coefficients and lower wear rates. Smaller grain size and fewer defects at 100 °C result in this behaviour [181].

#### 4.2.2. Composition

Composition of nanocoatings influences their tribological performance. Ni-W alloy coatings with tungsten contents ranging from 3 at % to 28 at % revealed that the addition of tungsten enhanced their wear resistance [182]. The enhancement in wear resistance was attributed to the increase in hardness, an effect based on Archard’s wear law [183]. Furthermore, it was observed that a reduction in grain size leads to increased hardness [182]. Nanocrystalline cobalt and cobalt–tungsten alloy coatings fabricated by pulse reverse electrodeposition method showed that Co-W nanocoatings outperformed pure Cu nanocoatings in terms of wear resistance. This was primarily due to the tungsten addition, which enhances hardness [184]. Unlike W addition in coatings, wherein an increase in W improves wear resistance, in Ni-Fe nanocoatings, the reduction in the Fe content improves the wear resistance of the coatings [178]. This contrast in wear performance is directly indicative of the importance of inherent properties of the alloying elements. While an increase in tungsten that has higher hardness improves wear resistance, an increase in softer Fe content reduces wear resistance.

#### 4.2.3. Grain Size

Grain size is inversely related to the yield strength of a material. Lower grain size increases yield strength, according to the Hall–Petch equation [185]. Nanocrystalline pure nickel coatings deposited by pulse electrodeposition having grain size (varying from 21 nm to 43 nm) on mild steel substrates showed that the sliding wear rate and coefficient of friction decreased as the grain size decreased. The wear rate lowered by a factor of five when the grain size was reduced from 195 nm to 21 nm, accompanied by a decrease in the coefficient of friction [186]. An enhancement in the wear resistance with reduction in grain size was observed in nanocrystalline nickel and nickel tungsten coatings, deposited on steel substrates [182]. In nanocrystalline pure nickel coatings, the wear rate decreased with the decrease in grain size, until grains reached a critical size ranging from 10 to 22 nm [187]. In Ni-Fe alloy coatings deposited on copper produced by pulse electrodeposition, the hardness and wear resistance were significantly enhanced due to the reduction in grain size of the nanocoatings [178].

### 4.3. Nanocomposite Coatings

Nanocomposite coatings are coatings that contain well-dispersed nano-sized components generally added to a matrix phase, which is usually polymeric, metallic or ceramic [188].

#### 4.3.1. Metallic Matrix Nanocomposite Coatings

Nanostructured Ni60-TiB_2_ composite coatings were deposited on steel substrates using the HVOF (high-velocity oxy-fuel) technique and their mechanical and tribological properties were compared to those of the micro-sized Ni60-TiB_2_ coatings, using ball-on-disc configuration [189]. The Ni60-TiB_2_ nanocomposite coating exhibited lower wear coefficient (i.e., less volume loss) than that of the micro-sized coating. This was due to the higher fracture toughness, higher hardness and higher strength of the nanocoatings. When compared to microcoatings, the fracture toughness and hardness of the nanostructured coatings were found to be 84% and 62% higher, respectively, due to their homogeneous microstructure and strengthening by grain refinement. Adhesive and mild abrasive wear were predominant wear mechanisms in nanocomposite coatings [189]. In h-BN/Ni60 and nano-Cu/h-BN/Ni60 laser cladded on Q235 steel [190], although the addition of h-BN and nano-Cu decreased the hardness of Ni60-based coatings, when compared to Ni60 and h-BN/Ni60 coatings, the nano-Cu/h-BN/Ni60 coatings experienced lower friction coefficient and wear rate in the temperature range of 25–500 °C. Both h-BN and soft copper provide a lubricating effect in nano-Cu/h-BN/Ni60 coatings, thereby lowering the friction coefficient and wear rate [190]. When compared to coatings without heat treatment and those treated for 2 h, the Ni-60/h-BN nanocoating deposited via laser cladding on 304 stainless steel substrates showed a low friction coefficient and low wear loss upon 1 h of heat treatment. This is due to the reduction in residual stress and increase in the fracture toughness induced by the heat treatment [191]. The addition of carbamide to the electrodeposition electrolyte (Watts bath) at a concentration of 10–15 g/L enhanced microhardness by up to 85% when compared to alumina-reinforced nickel nanocomposite coating (Ni-Al_2_O_3_) produced without carbamide. The wear rate drastically reduced in coatings containing carbamide, such that at a carbamide concentration of 15 g/L, the wear rate reduced to approximately 30% of that of the nanocomposite coating deposited without carbamide, as the addition of carbamide causes dispersion hardening [192]. 

**Figure 10:**
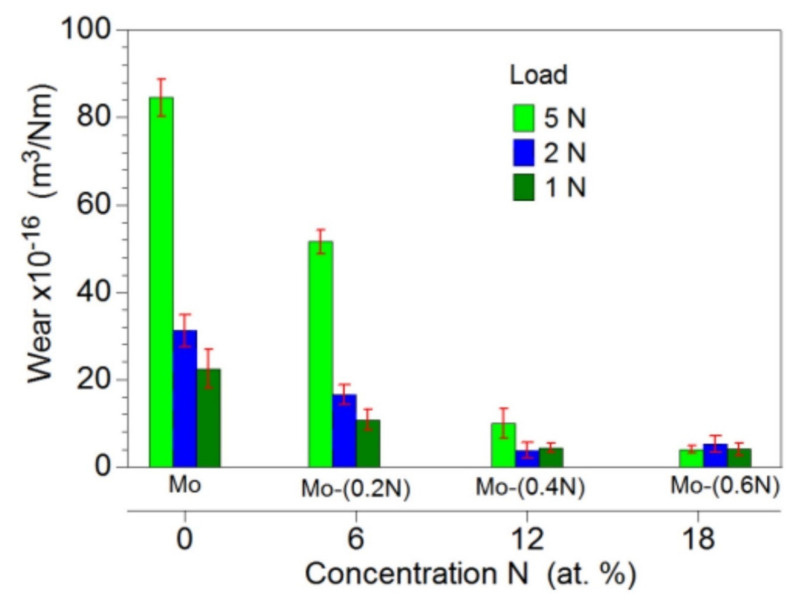
Wear of Mo-Mo_2_N coatings with varying concentrations of N [193].

Mo-Mo_2_N nanocomposite coating deposited on Ti-6Al-4V alloy by magnetron sputtering showed the dependence of its tribological behaviour on the nitrogen content present in the coatings [193]. With the increase in the nitrogen concentration in the coatings, the wear resistance increased. The best tribological performance, i.e., the lowest specific wear rate, was exhibited by the nanocomposite coating containing 18.6% nitrogen obtained at a nitrogen flow rate of 0.6 sccm (standard cubic centimetres per minute), as can be seen from Figure 10. The increase in nitrogen flow increases the nitrogen content in the coating, leading to refinement of columnar crystals [193].

**Figure 11:**
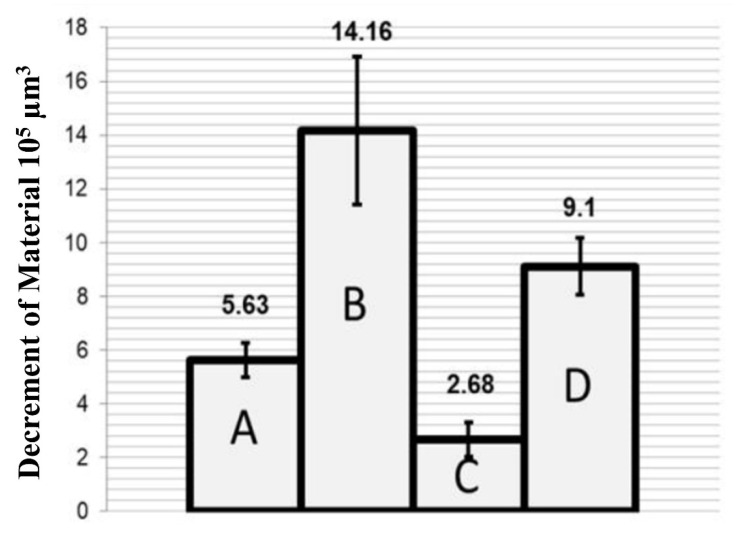
Material loss in (A) Zn coating at 1 N load, (B) Zn coating at 2 N load, (C) Zn/NP nanocomposite coating at 1 N load and (D) Zn/NP nanocomposite coating at 2 N load [194].

Zinc-based nanocomposite coatings comprising metal oxide nanoparticles (Zn/NP) electrodeposited on austenitic 316 L steel showed a considerable improvement in hardness and consequently showed a lower wear compared to plain zinc coatings. Figure 11 shows the material loss for the nanocomposite coatings [194]. 

#### 4.3.2. Polymer Matrix Nanocomposite Coatings

**Figure 12:**
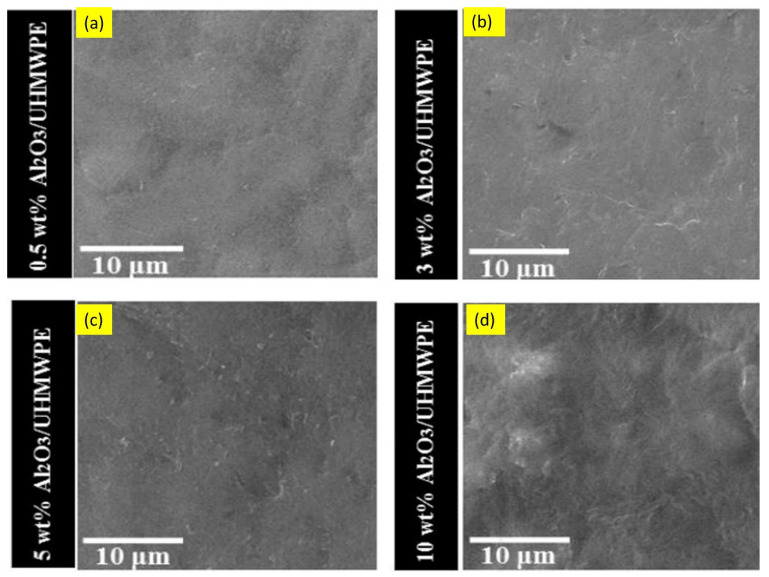
FESEM images for UHMWPE polymer coatings reinforced with (**a**) 0.5, (**b**) 3, (**c**) 5 and (**d**) 10 wt % nano-alumina particles [196].

**Figure 13:**
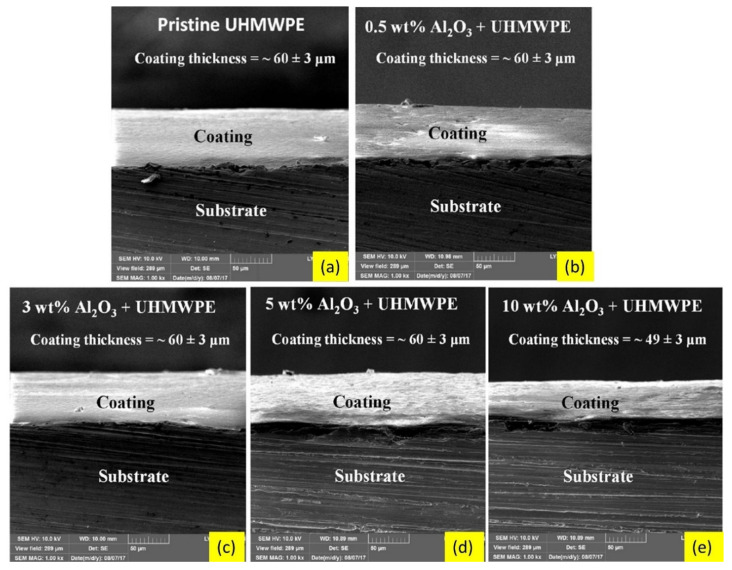
FESEM cross-sectional images of the UHMWPE coating/steel substrate system for the (**a**)pristine, (**b**) UHMWPE with 0.5 wt % Al_2_O_3,_ (**c**) UHMWPE with 3 wt % Al_2_O_3_, (**d**) UHMWPE with 5 wt % Al_2_O_3_, and (**e**) UHMWPE with 0.5 wt % Al_2_O_3_ nanocomposite coatings [196].

**Figure 14:**
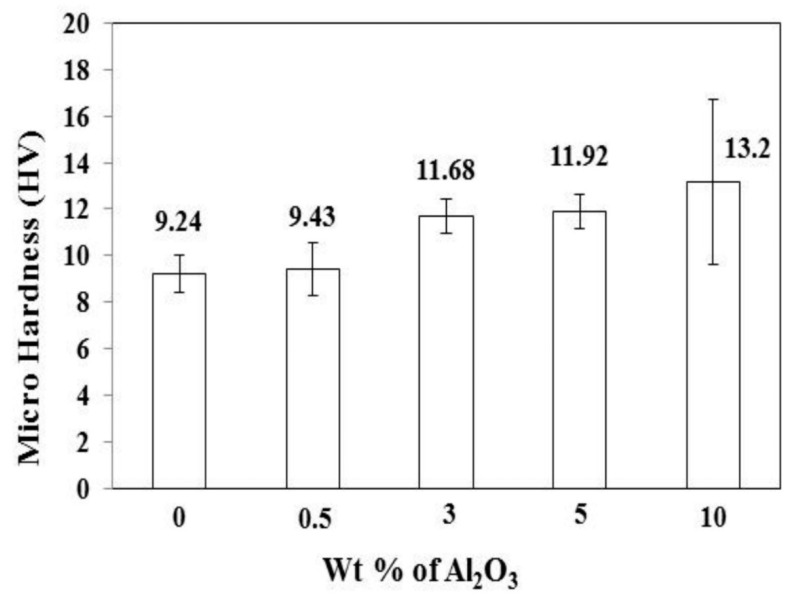
Microhardness of nanocomposite coatings (UHMWPE) with varying percentages of Al_2_O_3_ [196].

The incorporation of nanoparticles in polymer matrix nanocomposite coatings can significantly improve their wear resistance [195]. For example, the reinforcement of nano-alumina particles (Al_2_O_3_) to ultra-high molecular polyethylene (UHMWPE) coatings on steel substrates considerably enhanced microhardness and wear resistance, compared to coatings without alumina particles. Field emission scanning electron microscopy (FESEM) images of nanocomposite coatings with various alumina content and those of coating/substrate system are shown in Figure 12 and Figure 13, respectively. Nanocomposite coatings containing 3 wt % and 5 wt % alumina particles demonstrated excellent tribological performance, as they did not fail even after 250,000 sliding cycles, whereas the coatings containing 0 wt % and 0.5 wt % failed after 20,000 and 50,000 cycles, respectively, at the normal load of 12 N. This shows that 3 wt % and 5 wt % alumina particles are required to increase the hardness of the polymer coating and hence increase its wear resistance. The nanocomposite coating with 10 wt % alumina failed very early (after 4000 sliding cycles) due to non-uniform dispersion and aggregation of alumina particles. Figure 14 shows the variation in microhardness of nanocomposite coatings with varying weight percentages of alumina [196].

UHMWPE polymer nanocomposite coatings reinforced with 1 wt % graphene nanoparticles (GNPs) exhibited low friction and wear [120]. This behaviour was attributed to the lubrication effect produced by the sliding of graphene sheets. Polyurethane polymer matrix composite was reinforced with nano-TiC particles (1%, 2% and 3%) [197]. The addition of nano-TiC particles reduced the wear rate. The reduction in the wear rate was due to the enhanced hardness because of TiC particle addition. However, this improvement was only observed for up to a 2% addition of nanoparticles; additions >2% resulted in poor adhesion of the coating with the substrate and caused delamination of the coating from the substrate, which increased the wear [197].

#### 4.3.3. Ceramic Matrix Nanocomposite Coatings

Ceramic nanocomposite coatings are used in several engineering applications due to their high hardness and high wear resistance [198,199,200]. 

Ceramic matrix nanocomposite coatings developed by in situ reactive plasma spraying Al_2_O_3_-Fe_2_O_3_ composite powders were hardened by an in situ-generated metal phase. Compared to micro-Al_2_O_3_ monophase coatings, the nanostructured composite coatings demonstrated higher microhardness, toughness and anti-wear capabilities [201]. Laser surface-treated Al_2_O_3_-TiB_2_-TiN nanocomposite coatings were formed by two different routes, i.e., in situ and ex situ [200]. In the in situ technique, reinforcing phases TiB_2_, TiN and the matrix Al_2_O_3_ were synthesised from a mixture of Al, TiO_2_ and h-BN by laser-induced self-propagating high-temperature synthesis, and subsequently laser surface-alloyed on a low-carbon steel substrate. In the ex situ process, the composite constituents were directly laser surface-alloyed onto the substrate. When compared to ex situ coatings, the in situ Al_2_O_3_-TiB_2_-TiN nanocomposite coatings had better dispersion, fewer defects and higher wear resistance [200]. In AlMgB_14_-TiB_2_ nanocomposite coatings, oxidation of the TiB_2_ phase occurred. This oxide phase reacts with moisture to form a surface layer of boric acid, which reduced friction. Friction coefficient reduced to as low as 0.02 in combination with the high hardness of the mixed-phase. These coatings showed remarkable wear resistance and low friction. As boric acid formation was solely associated with the TiB_2_ phase, such coatings can be designed for specific applications by altering the AlMgB_14_ to TiB_2_ ratio [202]. 

Sliding wear properties of SiC-Al_2_O_3_ nanocomposite coatings produced on aluminium 6061 alloy substrates using plasma spraying [203] showed the influence of silicon carbide and alumina compositions on wear behaviour (applied normal load: 30 N; sliding velocity: 2 m/s). The wear resistance of all the coated samples was much higher compared to that of the uncoated surface. Furthermore, the substrate coated with the composite coating consisting of 50% SiC and 50% Al_2_O_3_ had the lowest wear rate of all the samples [203]. In a similar study, a ceramic coating produced by micro-arc-oxidation was deposited on an AZ91D magnesium alloy substrate [204]. The ceramic coating was composed of MgO and MgAl_2_O_4_ phases. To increase the wear resistance of the ceramic coating, silicon carbide (SiC) nanoparticles were incorporated into the coating. Reciprocating sliding wear tests were conducted on the coated surface using a GCr15 steel ball. The incorporation of SiC nanoparticles increased the wear resistance of the ceramic coating when compared to the coatings that did not contain SiC particles. This is due to the uniform distribution of SiC particles and the formation of a compact layer [204]. In magnesium alloys used for automotive applications, surface modification using rare earth compounds have been found to be effective in improving their surface mechanical and chemical properties [205]. In one such study, CeO_2_ nanoparticles, a rare earth compound, were incorporated during the deposition of aluminate-based PEO (plasma electrolytic oxidation) composite coatings on AM50 magnesium alloys. The influence of CeO_2_ nanoparticles on the sliding wear behaviour of the composite coating was examined against AISI 52,100 steel balls. The results showed that the CeO_2_ nanoparticles had a considerable effect on wear behaviour, particularly at loads of 5 N and 10 N. At these higher loads, the coatings without CeO_2_ nanoparticles failed, whereas the coatings with CeO_2_ nanoparticles worked remarkably well by enhancing the wear resistance of the AM50 magnesium alloy [205]. The enhanced wear resistance of the composite coating was attributed to the incorporation of ceria nanoparticles, which reduced the porosity in the coatings, thereby resulting in higher hardness [205].

**Figure 15:**
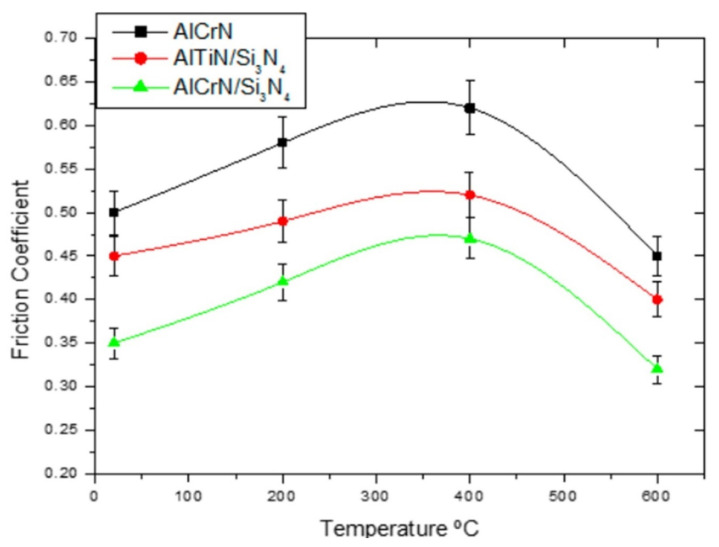
Friction coefficient of the AlCrN, AlTiN/Si_3_N_4_ and AlCrN/Si_3_N_4_ coatings at various temperatures [206].

**Figure 16:**
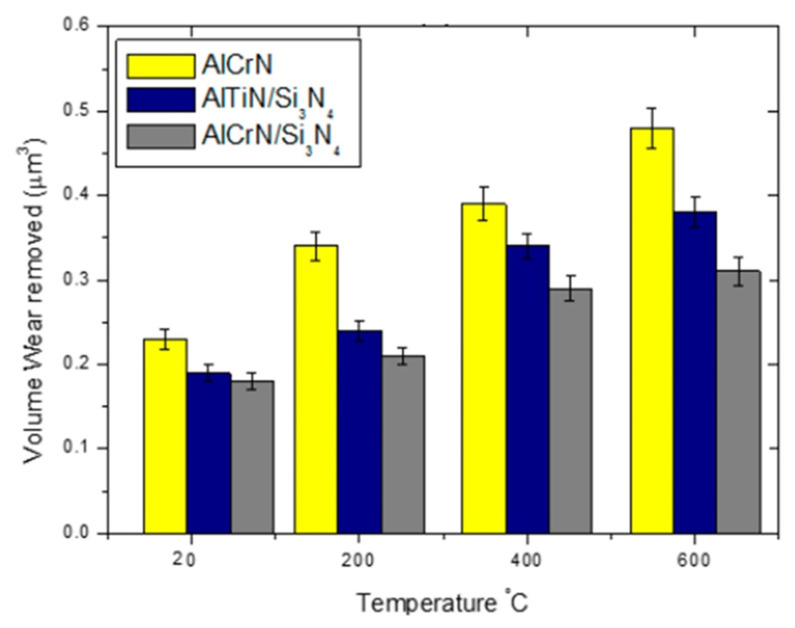
Volume wear for AlCrN, AlTiN/Si_3_N_4_ and AlCrN/Si_3_N_4_ coatings at various temperatures [206].

**Figure 17:**
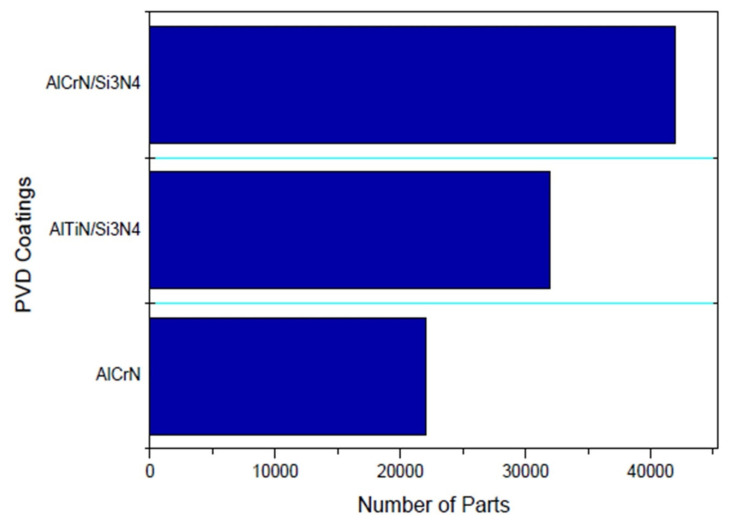
Number of parts produced by high-pressure die casting (HPDC) until defected parts are produced [206].

Using physical vapor deposition (PVD), two nanocomposite coatings (AlTiN/Si_3_N_4_ and AlCrN/Si_3_N_4_) with Si_3_N_4_ ceramic matrix were developed for an aluminium die casting tool [206]. Compared to other coatings, the frictional coefficient of AlCrN/Si_3_N_4_ nanocomposite coating was significantly lower (Figure 15), especially at high temperatures, along with decreased wear volume at all temperatures (Figure 16). Tool life clearly improved as a result of increased hardness of the coatings. When compared to AlCrN coating, the tool life of AlCrN/Si_3_N_4_ nanocomposite coated mould increased by 92% as a result of the increased hardness of the coatings (Figure 17) [206].

## 5. Biomimetic Approaches

Natural systems provide valuable insights to design engineering systems [207]. This trend can also be seen in the development of coatings [208]. Various aspects from natural systems guide the design and development of coatings/surfaces for functionalities such as hydrophobicity, improving interfacial adhesion, anticorrosion and reduction of friction/wear [209,210,211,212,213,214,215]. In this section, examples of biomimetic research works related to coatings for corrosion and tribological applications are presented. 

### 5.1. Nature Inspired Anticorrosion Coatings

Anticorrosion properties can be achieved by learning from nature via (i) hydrophobicity, (ii) surface texturing, (c) surface treatments and (iv) micro-alloying. Corrosion resistance can be improved by synthesising superhydrophobic coatings with surface features that mimic those of marine plants [216]. The morphology of water-repellent plants provides insights to develop effective functional coatings. Guided transportation of fluids in various living systems has given inspiration to develop coatings that restrict the transportation of corrosive media within coatings.

**Figure 18:**
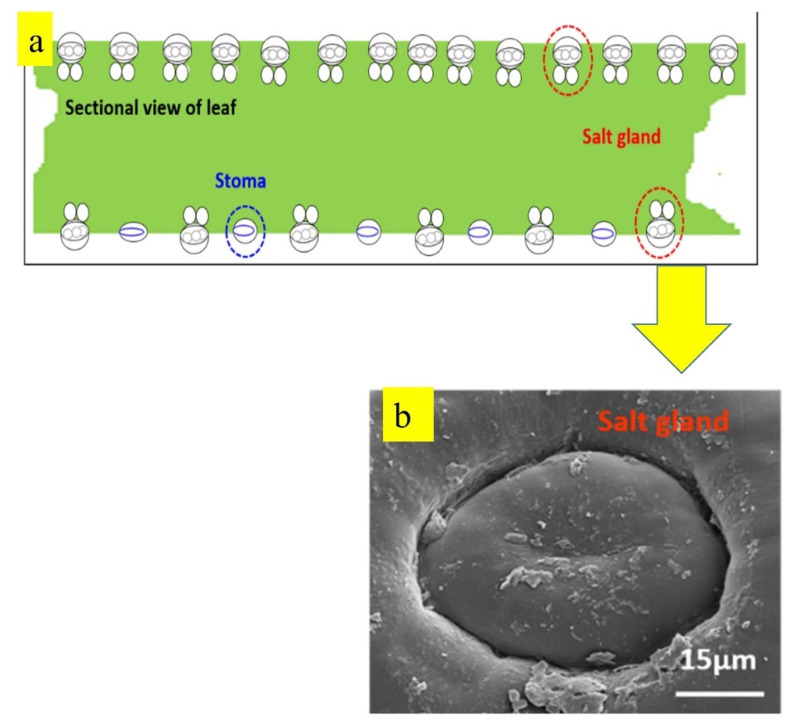
(**a**) Schematic showing the distribution of salt glands and stomata on a mangrove leaf that regulate salt transportation in and out of leaf. (**b**) SEM image of a salt gland of a mangrove leaf [221].

An anticorrosive coating that is superhydrophobic as well as self-healing was developed by mixing the matrix of polyvinylidene fluoride (PVDF) with nano-SiO_2_ and 2-mercaptobenzothiazole (MBT)-loaded halloysites (HNTs), which gave rise to a hierarchical columnar structure [217]. Two-dimensional materials such as graphene and graphene oxides have been added as fillers in coatings to restrict the flow of corrosive ions, thereby preventing/delaying the occurrence of corrosion [218]. A coating made of zinc phosphate by mimicking nepenthes pitcher plant was developed by a facile method, which exhibited better corrosion resistance when compared to the bare substrate [219]. It has been reported that ultrathin nanosheets of graphene sandwiched in between epoxy layers mimic the micro-nano structures of nacre and mussels, and can effectively obstruct galvanic corrosion [220]. Anticorrosion coatings inspired by marine mangrove leaves, doped with ion-selective resins and structures [221], have shown the capability to confine the movement of corrosive media at coating–metal interface. Figure 18 shows the distribution of salt glands on the surface of mangrove leaf that act as a guard to regulate salt transportation in and out of leaves [221]. 

### 5.2. Nature Inspired Tribological Coatings

Structural designs found in nature, such as textures of various animals (e.g., gecko feet, fish scales, chiton teeth, etc.) and plant structures (e.g., surface features on lotus leaves, pitcher plant, etc.), have inspired the development of coatings that can provide low friction and wear properties. 

Inspired by the structural and tribological behaviour of the hind leg femur-tibia joint in adult katydids (Orthoptera: Tettigoniidae), micro/nanopatterned surface coatings were developed, which reduced adhesive forces due to contact area minimisation [222]. Nano-wrinkled thin hard films (hard) on polymers (soft part), created by using titanium nitride (TiN) and a-C:H on ultra-soft, highly viscoelastic thermoplastic polyurethane (PU), which mimicked human skin, showed a low coefficient of friction [223]. 

Natural systems and processes are intrinsically complex, and thus are difficult to replicate. Nevertheless, there exists an immense scope for further exploration of underlying principles of nature that can be utilised to develop nanocoatings with superior performance for corrosion and tribological applications.

## 6. Application Areas

Globally, industries encounter enormous economic losses due to chemical degradation of engineering surfaces by corrosion and mechanical damage by tribological issues. Nanostructured coatings provide an effective route to combat corrosion, friction and wear issues by surface protection, without resorting to the modification of bulk materials. Thus, nanostructured coatings have extensive applications. 

**Figure 19:**
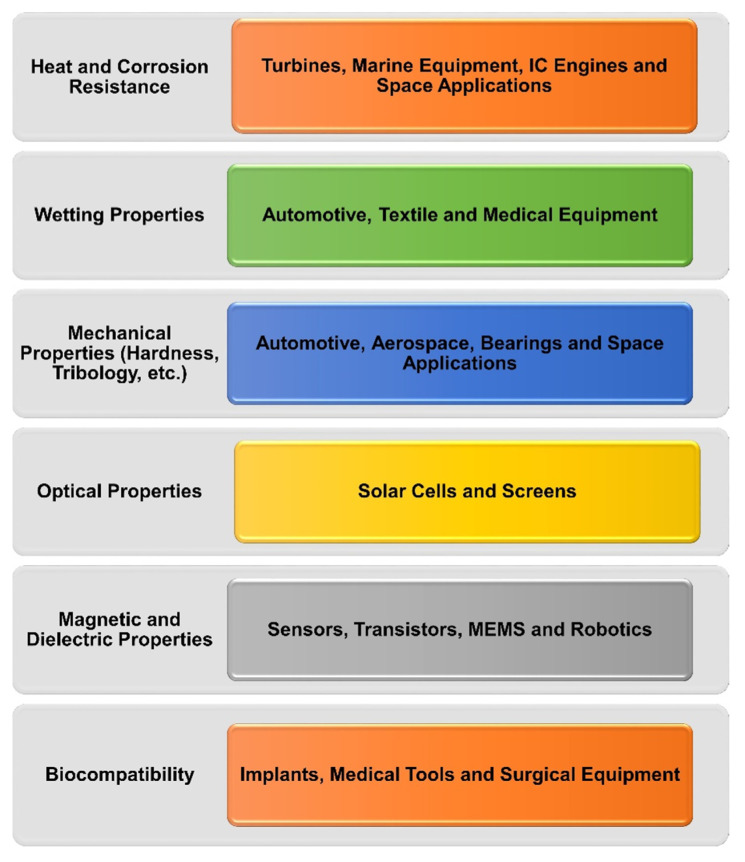
Application areas of nanostructured coatings [225].

Application areas of nanostructured coatings in various engineering systems include marine, aerospace, automotive, medicine (orthopaedic, dental), sports, construction, defence, energy, food packaging, etc. According to a report [224], the nanostructured coatings market is expected to rise by ~20% in 2020–2030. Prominent application areas of nanostructured coatings are given in Figure 19 [225]. Target properties, i.e., functionalities expected from nanostructured coatings for these application sectors, are also given in the figure.

## 7. Challenges in Developing Nanostructured Coatings

The development and synthesis of nanostructured coatings for anticorrosion and desired tribological performance have practical challenges, such as (i) selection of the right nanocoating material, specifically suitable for a given substrate and a given operating environment (e.g., ease of deposition, which becomes more challenging for a substrate having complex geometry and variation in size, adhesion to the substrate, etc.); (ii) selection of the right reinforcement/filler, most suitable for a given coating material and application (e.g., mechanical compatibility, thermal mismatch with matrix material, etc.); (iii) chemical complexity in synthesising nanocoatings; (iv) cost and time effectiveness of developing and deploying nanocoatings; (v) capability to apply nanocoatings over large surface areas; and (iv) long-term performance and maintenance.

## 8. Summary and Recommendations

This paper offers a comprehensive review on nanostructured coatings and nanocomposite coatings (ceramic coatings, metallic coatings and nanocomposite coatings with metal and polymer matrices), their synthesis, corrosion behaviour and tribological performance. The development of coatings for corrosion and tribological applications is a challenging endeavour given that several factors influence the properties and performance of coatings (coating material, composition, synthesis method, processing parameters, grain size, operating environment, additives, reinforcements/fillers, contact conditions, etc.). Issues such as the selection of coating material/reinforcements, chemical complexity, and cost and time effectiveness in synthesising coatings make the development of effective coatings even more challenging. Biomimetic coatings have shown promising potential for anti-corrosion and tribological applications. Developed nanostructured coatings and nanocomposite coatings have shown excellent performance at a laboratory scale; however, their translation to real-world applications is yet to be realised. 

Nanostructured coatings have potential applications in various engineering sectors, including marine, space/aerospace, automotive, robotics, medicine (e.g., orthopaedic, dental), sports, structure/architecture, defence, energy systems, etc. To realise the full applicative potential of coatings, the key issues that should be addressed are (i) strategies to improve interfacial adhesion between coatings and substrates, (ii) the development of low-cost processes for coating preparation, (iii) valuable insights from natural systems for coatings design and (iv) the development of mathematical models of coating processes and their effect on corrosion and tribological properties. This would facilitate the optimisation of coating processes and properties, prior to their real-world deposition. Additive manufacturing technologies can play a key role in the advancement of nanostructured coatings and their applications [27,226].

## Figures and Tables

**Table 1:** Coating preparation methods and their process parameters.

No.	Coating Preparation Method	Process Parameters	Coating Material
1.	CVD (a) Thermal-Enhanced Atomic Layer Deposition (b) Plasma-Enhanced Atomic Layer Deposition	Target material Temperature Deposition time Surface treatments Size of nanoparticles	Metallic, Ceramic
2.	PVD (a) Filtered Cathodic Arc Deposition (b) Reactive Sputter Deposition (c) DC Magnetron Sputtering	Target composition Substrate temperature Pre-etching Working pressure Power Magnet configuration	Nanocomposite, Metallic
3.	Spray Coating High-Velocity Oxy-Fuel (HVOF)	Temperature Powder feed rate Distance between gun and substrate Fuel/oxygen ratio	Ceramic, Metallic Nanocomposite
4.	Sol-Gel Process	Catalyst nature Hydrolysis ratio Temperature pH Initial material to produce sol	Polymer Matrix Nanocomposite
5.	Electrodeposition (a) Pulse Jet Electrodeposition (b) Reverse Pulse Electrodeposition (c) Direct Current Electrodeposition	Bath composition Bath temperature Time of electrodeposition Additives pH Current density Current (DC or pulse)	Metallic, Metallic Matrix Nanocomposite
6.	Laser Cladding	Cooling rate Laser power density	Metallic, Ceramic

## Data Availability

Not applicable.
